# Educational interventions for cervical cancer prevention: a scoping review

**DOI:** 10.1590/0034-7167-2023-0018

**Published:** 2023-11-27

**Authors:** Josiane Montanho Mariño, Lailah Maria Pinto Nunes, Yasmin Cardoso Metwaly Mohamed Ali, Leonardo do Carmo Tonhi, Marina de Góes Salvetti

**Affiliations:** I Universidade de São Paulo. São Paulo, São Paulo, Brazil; II Universidade Federal do Amazonas. Coari, Amazonas, Brazil; III Instituto Nacional do Câncer. Rio de Janeiro, Rio de Janeiro, Brazil

**Keywords:** Uterine Cervical Neoplasms, Mass Screening, Papanicolaou Test, Health Education, Primary Health Care., Neoplasias del Cuello Uterino, Tamizaje Masivo, Prueba de Papanicolaou, Educación en Salud, Atención Primaria de Salud., Neoplasias do Colo do Útero, Programas de Rastreamento, Teste de Papanicolaou, Educação em Saúde, Atenção Primária à, Saúde.

## Abstract

**Objectives:**

to identify, map and describe characteristics of educational interventions for cervical cancer prevention in adult women.

**Methods:**

a scoping review conducted on eleven databases and the gray literature, including studies that described educational interventions aimed at preventing cervical cancer in adult women.

**Results:**

thirty-three articles with 151,457 participants were analyzed. The most used educational strategies were participatory discussions and educational leaflets. Most of the interventions took place in a single session, ranging from 40 to 60 minutes. The most used theoretical model in interventions to improve women’s compliance with Pap smear was the Health Belief Model.

**Conclusions:**

group discussions, lectures and educational brochures can increase knowledge and reduce barriers to cervical cancer prevention. Theory-based and culturally sensitive interventions can have a positive impact on women’s health.

## RESUMO

**Objetivos:**

identificar, mapear e descrever as características de intervenções educativas para a prevenção do câncer cervical em mulheres adultas.

**Métodos:**

revisão de escopo, conduzida em onze bases de dados e na literatura cinzenta, incluindo estudos que descreveram intervenções educativas voltadas à prevenção do câncer cervical em mulheres adultas.

**Resultados:**

33 artigos com 151.457 participantes foram analisados. As estratégias educativas mais utilizadas foram as discussões participativas e folhetos educativos. A maior parte das intervenções ocorreu em sessão única, com variação de 40 a 60 minutos. O modelo teórico mais utilizado nas intervenções para melhorar a adesão das mulheres ao exame Papanicolau foi o Modelo de Crenças em Saúde.

**Conclusões:**

discussões em grupo, palestras e folhetos educativos podem aumentar o conhecimento e reduzir barreiras para a prevenção do câncer do colo do útero. Intervenções baseadas em teoria e culturalmente sensíveis podem ter impacto positivo na saúde das mulheres.

## RESUMEN

**Objetivos:**

identificar, mapear y describir las características de las intervenciones educativas para la prevención del cáncer de cuello uterino en mujeres adultas.

**Métodos:**

revisión de alcance, realizada en once bases de datos y en la literatura gris, incluyendo estudios que describían intervenciones educativas dirigidas a la prevención del cáncer de cuello uterino en mujeres adultas.

**Resultados:**

se analizaron 33 artículos con 151.457 participantes. Las estrategias educativas más utilizadas fueron las discusiones participativas y los folletos educativos. La mayoría de las intervenciones se realizaron en una sola sesión, con una duración de 40 a 60 minutos. El modelo teórico más utilizado en las intervenciones para mejorar la adherencia de las mujeres al Papanicolaou fue el Modelo de Creencias de Salud.

**Conclusiones:**

las discusiones grupales, las conferencias y los folletos educativos pueden aumentar el conocimiento y reducir las barreras para la prevención del cáncer de cuello uterino. Las intervenciones basadas en teorías y culturalmente sensibles pueden tener un impacto positivo en la salud de las mujeres.

## INTRODUCTION

Cervical cancer (CC) is the fourth most common cancer among women in the world, with an estimated 604,000 new cases and 342,000 deaths in 2020^(1)^. About 90% of new cases and deaths occur in lowand middle-income countries^(1)^. CC mortality can be reduced through regular examinations and early treatment^(2)^. However, inadequate infrastructure and funding difficulties to implement CC control strategies are limiting in developing countries^(3)^.

Periodic Pap smear testing continues to be the most adopted strategy for the screening of precursor CC lesions^(4)^. The World Health Organization (WHO) recommends performing Papanicolaou smear every three years in women aged 25 to 64 years after two negative tests with an annual interval^(5)^.

Awareness of the benefits of CC screening, diagnostic methods, the importance of early follow-up and treatment are among the main factors that encourage women to perform Pap smears^(6)^. Higher return rates are expected for well-informed women, who more easily adhere to the recommendations of health professionals and find innovative ways to deal with the disease, being less susceptible to complications^(7)^.

Several studies have tested educational strategies to strengthen the attitudes, practices and knowledge of women candidates for CC screening as well as to reduce barriers to compliance with these programs^(8-14)^. Among these theoretical models, we can mention the Health Belief Model (HBM)^(15)^, the Motivation Protection Theory (MPT)^(16)^ and the Social Cognitive Theory (SCT)^(17)^.

Despite CC screening campaigns, compliance remains low in most developing countries. Thus, it is important to know the educational intervention characteristics that have been used to improve CC prevention.

## OBJECTIVES

To identify, map and describe the characteristics of educational interventions for CC prevention in adult women.

## METHODS

This is a scoping review conducted according to the JBI methodology, described in the JBI Reviewer Manual 2020^(18)^, and Preferred Reporting Items for Systematic reviews and Meta-Analyses extension for Scoping Reviews (PRISMA-ScR) recommendation^(19)^, seeking to answer the following research question: what is the scientific evidence on educational interventions for CC prevention in adult women? The protocol for this review was registered on the Open Science Framework (OSF) platform and can be consulted at the link: osf.io/4zgex.

The search strategy organization used the acronym PCC, with P for population, C for concept and C for context^(18)^. Population consisted of studies involving adult women (age ≥18 years) with no previous diagnosis of CC. The key Concept of this review was composed of studies that detailed the characteristics of educational interventions aimed at preventing CC (elements, mode of delivery, dose, educational materials, educational content, type of theory). Context was primary care, hospitals, clinics, community centers, schools or churches.

The search strategy aimed to locate published and unpublished studies, and occurred in two stages. In the first one, a search was carried out in the MEDLINE and Cumulative Index to Nursing and Allied Health Literature (CINAHL) databases, followed by the analysis of title and abstract words and study descriptors with the terms as follows: “Cervix cancer; cervical cancer; Uterine Cervical Neoplasms; screening; Prevention and control; Papanicolaou test; Pap test; Pap smear; education; intervention”.

In the second stage, a new search using all identified keywords and descriptors was carried out in the previously described databases. To develop the search strategy, we had the help of an experienced librarian. The searches used modified controlled vocabulary for each database.

Data collection took place between June 7 and August 14, 2022, in the PubMed/MEDLINE, Scientific Electronic Library Online (SciELO), PsycINFO, Cochrane Library databases, through Virtual Health Library (VHL) portal; in the Latin American and Caribbean Literature in Health Sciences (LILACS) and Nursing Database (BDENF) databases, through the Coordination for the Improvement of Higher Education Personnel (CAPES - *Coordenação de Aperfeiçoamento de Pessoal de Nível Superior*) portal; and in the CINAHL, Web of Science, Embase, Scopus databases. Unpublished studies were searched in the gray literature (Google Scholar, Catalog of Theses and Dissertations CAPES - Brazil and Brazilian Registry of Clinical Trials - ReBEC, Digital Library of Theses and Dissertations (BDTD).

This review included qualitative and quantitative studies focusing on educational interventions aimed at preventing CC in adult women. Qualitative studies of any theoretical and methodological approaches were considered as well as studies published in English, Spanish or Portuguese, with no date limit.

Intervention studies that addressed other types of cancer (breast cancer, colon cancer, colorectal cancer and others), studies that used only an invitation letter or reminder for Pap smear, without educational content based on literature or theory, were excluded. Systematic or integrative reviews and study protocols were also excluded, as they did not present in detail the elements of the educational intervention (method of delivery, dose, educational materials, intervenor). Disagreements were resolved by discussion between the two reviewers and, when necessary, by a third reviewer.

All titles and abstracts retrieved in searches were pooled in the Mendeley^®^ reference management database for identification and exclusion of duplicates. For selection and assessment of sample studies, the Rayyan software was used^(20)^. Study pre-selection was performed by reading the title and abstract by two reviewers independently, based on established inclusion criteria.

Data extraction from the studies included in the review was performed using a standardized data extraction tool (Appendix 2). General data extracted included data on the authors, year of publication, objectives, methods, study design, outcomes, target population characteristics (age, race/ethnicity), intervention characteristics (educational strategies, mode of delivery, dose, intervenor), theoretical structure, type of intervention (individual, group or multicomponent) and main results of the intervention.

## RESULTS

**Figure 1:**
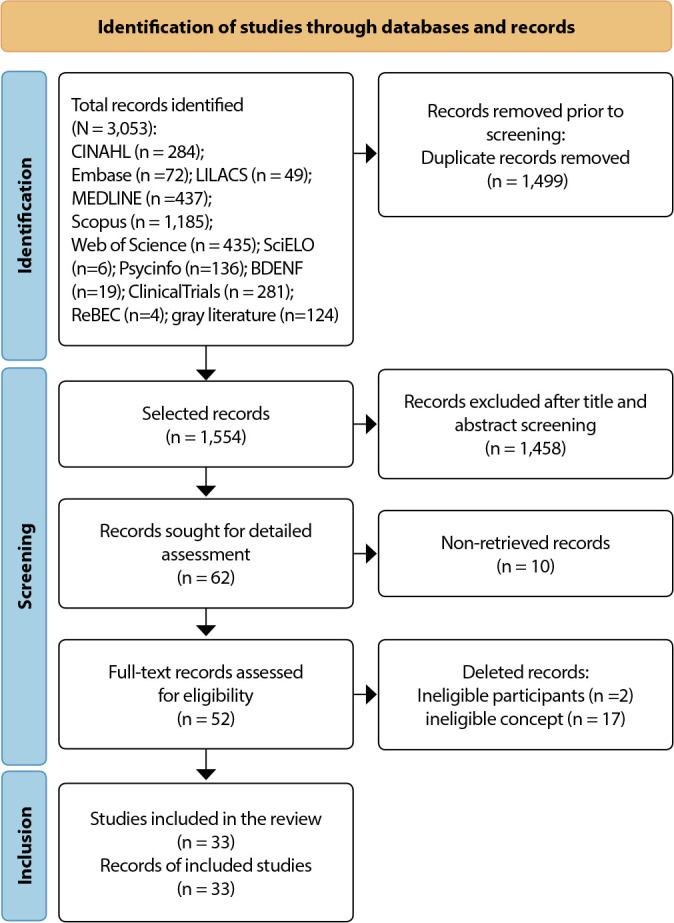
Study search flowchart based on Preferred Reporting Items for Systematic reviews and Meta-Analyses extension for Scoping Reviews, Coari, Amazonas, Brazil, 2022

Figure 1 expresses the search and selection results presented by the PRISMA-ScR flowchart^(19)^. After reading, some studies were excluded for not describing the elements of the intervention and the type of theory used, leaving 33 articles for analysis.

In the analyzed studies, the most used design was the quasi-experimental, which appeared in 18 studies (54.5%)^(8,11-13,21-34)^. Twelve studies were Randomized Clinical Trials (RCTs)^(7,10,12,35-43)^; two were pre-test and post-test pilot studies^(14,44)^; and one was mixed method study^(10)^. Most of studies were carried out in the USA (36.3%)^(11,14,27,29,34,36-38,41-44)^, Iran (30.3%)^(7,21,24,31-32,39,45)^ and Turkey (9.1%)^(10,25-26)^ and published between 2007 and 2022 (Chart 1).

**Chart 1:** Description of the main characteristics of analyzed studies

Code/author/year/country	Design/sample/theory	Population characteristics	Intervention characteristics
A1^(40)^ Mboineki, et al. 2022 Tanzania	RCT n=88 Theory of Planned Behavior, HBM and Diffusion of Innovation Theory	Women aged 21 to 50, who have never had a preventive exam, without a previous history of cancer. Never received health education about CC and not being pregnant.	Peer-Led Navigation (PLNav), a single session lasting two hours over 6 months. The intervention included health education about CC and CC screening, advice to women who did not undergo screening according to scheduled appointments, navigation care.
A2^(9)^ Hosseini et al. 2022 Iran	Quasi-experimental study n=202 BASNEF Model	Women aged 20 to 49, sexually active, not pregnant, with at least elementary school education.	Educational program about CC applied in 14 sessions of 40-60 minutes, with lectures, participatory discussion, questions and answers, brainstorming.
A3^(28)^ Lima 2017 Brazil	Quasi-experimental study n=524 Motivational Interviewing: Evoke-Provide-Evoke Model	Women between 25 and 64 years of age, who have started sexual activity, inadequate frequency of examinations and have their mobile or landline telephone numbers in their medical records.	Telephone intervention in a single 15-minute session. Brief explanation about CC and its risks, purpose of Pap smear test, importance of Pap smear test frequency, pre-examination care and the return for the result.
A4^(12)^ Mirzaei-Alavijeh et al. 2014 Iran	RCT n=120 Theory of Planned Behavior	Women aged 35 to 64, married and attending health centers	Intervention in four weekly sessions lasting 45-60 minutes each (one lecture and three group discussions) on CC, related factors and the role of Pap smear.
A5^(35)^ Abu et al. 2020 Ethiopia	RCT n=2203 HBM	Women aged 30 to 49 who sought care at maternal and child health clinics but who were never screened for CC.	The intervention consisted of a brief individual conversation about CC and benefits of screening lasting 5 to 10 minutes provided by nurses. At the end, the participants received a leaflet containing information on the topics addressed.
A6^(14)^ Ramaswamy et al. 2015 USA	Pilot study n=7 Bourdieu’s Theory of Social Transformation and Feminist Theory	Women aged ≥18 years, incarcerated, who have never had a Pap smear and who have not been diagnosed with cancer.	Educational intervention on sexual empowerment consisted of five two-hour sessions, over the course of a week, including group discussions and delivery of health agency leaflets by a nurse and physician.
A7^(8)^ Ernawati et al. 2021 Indonesia	Quasi-experimental study n=100 HBM	Married women, aged ≥18 years, able to communicate in Indonesian (national language), regardless of whether they can read or not.	Educational intervention with one session lasting one hour, including lecture, space for questions and answers, group discussions and booklets on CC and CC screening.
A8^(30)^ Luque et al. 2017 Georgia	Quasi-experimental study n=90 SCT and Popular Education Theory	Women ages 21 to 65, rural immigrants, Latinas who have not had a Pap smear in 2 years or more.	*Salud es Vida* group intervention, with an introduction to CC with video and flipchart as well as dialogue to explore barriers to health care. Sessions of approximately 3 hours, with an average of 7 participants per session.
A9^(43)^ Thompson et al. 2017 USA	RCT n=443 SCT	Women age 21 to 64 of Latino ethnicity residing in the Yakima Valley who have not had a Pap smear in the past 3 years and have not had a prior hysterectomy.	Low-intensity intervention: culturally appropriate video in Hispanic language. High-intensity intervention: educational session with a health worker at home (watching the video with the health worker, appointment to perform or schedule a Pap smear).
A10^(13)^ Pirzadeh et al. 2012 Iran	Quasi-experimental study n=70 HBM	Married women who have never had a Pap smear.	Group interventions with three sessions of 45 to 60 minutes in one week. Information about CC and films of cancer patients. Group discussion on the benefits and barriers to having Pap smear with films about the steps of the test.
A11^(11)^ McDonough et al. 2016 USA	Quasi-experimental study n=5211 HBM and SCT	Participants had to be 18 or older and identify as Latinas.	Intervention in a single two-hour session, offered over four years. Lectures with group discussions using flipchart and bilingual educational brochures listing local resources where participants could obtain a free or low-cost Pap smear test.
A12^(10)^ Koç et al. 2019 Turkey	RCT n=156 PRECEDE-PROCEED Conceptual Model	Women taking courses at the community training center, literate with no history of cancer and no previous training in CC or HPV.	Focus group discussions with 6 to 10 individuals on quality of life, CC history, behaviors, lifestyle and beliefs. The women received 3 sessions of 60 minutes on cancer, HPV infection, healthy lifestyle and behaviors, including healthy nutrition, physical activity and weight management.
A13^(21)^ Daryani et al. 2016 Iran	Quasi-experimental study n=120 HBM	Women aged 20 to 65 years, married for at least 6 months.	The group intervention was carried out in two sessions of 1 hour and 30 minutes. The first session included understanding CC, risk factors, symptoms and prevention. The second session included understanding the Pap smear. The teaching methods involved practical exhibitions, films, lectures, questions and answers, and a pamphlet based on the content of other media.
A14^(27)^ Lee et al. 2015 USA	Quasi-experimental study n=30 Fogg Behavior Model	Korean American women aged 21 to 29 without prior receipt of a Pap smear.	Intervention in seven sessions, with questions and answers about information about CC, Pap smear, accessibility to health care, cultural barriers, availability of local clinics, cost of Pap smear, testimony from a Korean American woman who experienced Pap smear, and testimony from a CC survivor.
A15^(29)^ Love et al. 2009 USA	Quasi-experimental study n=498 SCT	Thai women aged 18 and over.	Single session. 7-minute video for small groups of participants and discussion about their knowledge, attitudes and beliefs regarding Pap smear and CC.
A16^(41)^ Mishra et al. 2009 USA	RCT n=398 Structure of Health Behavior and Freire’s Pedagogy of Empowerment	Samoan women, age 20 years or older, no self-reported history of obtaining a Pap smear within the last two years, no history of CC, no history of hysterectomy, and staying in the territory for the duration of the study (about six months).	Three two-hour weekly educational sessions, with hands-on exhibits, CC lectures and space for questions and answers. A total of 20 groups with 8 to 14 women were assisted.
A17^(7)^ Samami et al. 2021 Iran	RCT n=120 HBM	Women aged 21 to 65 years, with no history of uterine surgeries and hysterectomy.	Group intervention with two 90-minute sessions. Practical presentation, film, lecture, questions and answers. The first session was about CC, prevention methods and how to perform Pap smear. The second session was about women’s attitude, knowledge and role towards Pap smear.
A18^(38)^ Calderón-Mora et al. 2022 USA	RCT n=500 HBM, Theory of Planned Behavior and SCT	Women ages 21 to 65, who have a Pap smear, are uninsured or underinsured, have no history of CC or hysterectomy, and have a Texas address.	Single-session group intervention (17-minute video on Pap smear, discussion of barriers to screening, followed by narration of infographics with guidance on screening and overcoming barriers). A 20-minute flipchart presentation was also used to reinforce the information.
A19^(23)^ Fang et al. 2007 Korea	Quasi-experimental study n=102 HBM and SCT	Korean women from two community organizations, aged 18 years or older, without a diagnosis of CC and a Pap smear in the past 6 months.	Two-hour educational session with lectures, discussions, navigation training, exhibition of cultural videos, role-play use and behavioral skills test. The educational session focused on CC and tips for action and strategies to overcome barriers.
A20^(33)^ Thompson et al. 2014 Mexico	Quasi-experimental study n=162 Principles of Mapping Intervention	Hispanic women, residents of border counties in New Mexico, ages 29 to 80, who have not had a Pap smear in the past 3 years.	The activity was individual, delivered by a CHW at home and consisted of one session. The intervention period was for 12 months. Intervention materials included a PowerPoint presentation that illustrated and described Pap smear, CC, and HPV. Color images and a video of a Pap smear were included in the presentation.
A21^(25)^ Guvenc et al. 2013 Turkey	Quasi-experimental study n=2,500 HBM	Women at least 21 years of age, not having a previous diagnosis of gynecological cancer, literate, sexually active currently or in the past, not having a Pap smear in the last 12 months, not being in the second or third trimester of pregnancy, nor in the postdelivery of 3 months and available for telephone contact.	Educational flyers with invitation to Pap smear; telephone interview: information on the topics in the booklet and invitation to perform the Papanicolaou test; face-to-face interviews: conducted at home to inquire about the reason(s) related to not participating in the free CC screening after two invitations.
A22^(24)^ Ghahremani et al. 2016 Iran	Quasi-experimental study n=420 Protection Motivation Theory	Married, non-pregnant women, who had never had a Pap smear, did not have CC, had no history of hysterectomy surgeries.	CHWs trained the women under their cover in person using educational pamphlets during 3 sessions over 21 days.
A23^(22)^ Drokow et al. 2021 Africa	Quasi-experimental study n=600 HBM and Transtheoretical Model	Ghanaian residents over 18 years of age, mentally healthy, not deaf or mute, with no previous history of HPV vaccination, cell phone or tablet carrier.	Three 3 sessions lasting 15 minutes. Educational videos about CC, HPV, Pap smear delivered by a nurse and a CHW. The video has been played twice for clarity. This was done every 2 months until the end of the 6-month intervention period.
A24^(34)^ Wang et al. 2010 USA	Quasi-experimental study n=134 HBM and SCT	Asian Chinese women, low-income, uninsured, no history of CC.	Two small group sessions led by trained Chinese community health educators. Specific syllabus focused on CC risk factors, prevalence and benefits of screening and early detection. Participants received help from the health professional to schedule appointments (nurse navigator). Handouts on CC and Pap smear and a video in Chinese on the subject were also presented.
A25^(36)^ Byrd et al. 2013 USA	RCT n=613 Mapping intervention, SCT, HBM, Transtheoretical Model and Rational Choice Theory	Self-declared women of Mexican origin aged 21 years or older, with no previous history of cancer, no hysterectomy, and no CC screening in the past 3 years.	AMIGAS intervention included a video drama using models to discuss barriers and facilitators to CC screening, a flipchart reviewing video information, games and activities, including a set of cards to understand a woman’s stage of change, in addition to a contract sheet titled “my promise”.
A26^(44)^ Fleming et al. 2018 USA	Pilot study n=60 SCT and HBM	Hispanic/Latino women, able to speak and read Spanish or English, aged 21-70.	Intervention with six 75-minute meetings. Group discussions, average of 10 participants per meeting. Community educators provided education using the CC educational resource as a structured and organized way of delivering content.
A27^(26)^ Kurt 2019 Turkey	Quasi-experimental study n=134.704 HBM	Women aged 30-65 (based on national CC screening age group), are or have been sexually active, able to speak, read and understand the Turkish language.	Brochure + education group: individual training on the importance of CC and Pap smear; leaflet only group: participants were asked to read the educational leaflet; Invitation-only group: Participants in this group were invited to receive a screening without additional training or an educational brochure.
A28^(32)^ Shobeiri et al. 2018 Iran	Quasi-experimental study n=330 HBM	Women over 18 years old, attending premarital education classes, wanting to get married for the first time, or divorced women wanting to remarry.	Intervention with two group sessions of 45-60 minutes/week. The sessions were led by a professor of medical sciences, including lectures on Pap smear, group discussions, questions and answers. Educational leaflets were handed out to participants at the end.
A29^(45)^ Khani et al. 2021 Iran	Interventional and prospective experimental study n=300 HBM and Theory of Planned Behavior	Women married for at least 6 months, not pregnant and without a history of cancer and/or hysterectomy.	Eight 50-minute educational sessions, once a week, with group discussions, brainstorming, Q&A and film screenings. At the end, a booklet and an educational CD were delivered. An educational message about the importance of CC prevention and screening behaviors was sent to the subjects each week and a telegram group was formed.
A30^(42)^ O’Brien et al. 2010 USA	RCT n=120 HBM	Hispanic women aged 18 to 65.	Intervention applied by CHW with two 3-hour sessions. Presentation of information about CC, delivery of pamphlets and educational booklets to groups of 4 to 10 women.
A31^(31)^ Parsa et al. 2017 Iran	RCT n=80 HBM	Married women, aged between 18 and 60, residing in the village for at least 2 recent years, no hysterectomy, no history of CC.	Group counseling intervention applied by rural CHWs in three sessions of 45-60 minutes with an interval of one week and a capacity of 10 people per session, using posters and pamphlets.
A32^(39)^ Malmir et al. 2018 Iran	RCT n=152 Protection Motivation Theory	Age over 20 and residing on the Kermanshah bank, not having a CC diagnosis and being married or sexually active.	Intervention with lectures on CC, group discussions and question-answers, pamphlets and a booklet were delivered by women from the region to participants, after each educational session. The activity took place in five sessions lasting 45 minutes for four weeks.
A33^(37)^ Calderón-Mora et al. 2020 USA	RCT n=300 HBM, Rational Choice Theory and SCT	Women between 21 and 65 years old, who have not had a Pap smear in the last 3 years, residents of El Paso or Hudspeth County, uninsured, no history of hysterectomy and/or CC, income >200% of the federal poverty level, or not enrolled with the Texas State Department of Health Services.	Intervention with group and individual discussions about barriers to screening and interactive dialogue. Participants received education with identical content from the AMIGAS project, with 75 minutes in the individual arm and 90 minutes in the group arm. Flipchart, message cards, body diagrams, action plan worksheet, resource sheet and information leaflets were used.

*CC - Cervical Cancer; HBM - Health Belief Model; RCT - Randomized Clinical Trial; CHW - Community Health Worker; BASNEF - Beliefs, Attitudes, Subjective Norms and Facilitating Factors; PRECED- PROCEED - Predisposing, Reinforcing and Enabling Constructs in Educational Diagnosis and Evaluation-Political, Regulatory and Organizational Constructs in Educational and Environmental Development; AMIGAS - Helping Women with Information, Guidance and Love for their Health; SCT - Social Cognitive Theory. CCS - Cervical cancer screening.*

The population of most of analyzed studies consisted of women aged 21 to 65 years, immigrants, Hispanic/Latina, and from rural environments, with a history of low adherence to Pap smear, no previous history of CC, married, non-pregnant, who had never received health education about CC and without health insurance. The total sample size in this review was 151,457 (Chart 1).

Regarding the context, most of studies were carried out in community health centers (n=19)^(7-9,11-13,21,24,28,30-33,35-36,38,40,43,45)^, followed by community centers (n=5)^(10,23,29,34,44)^, churches (n=4)^(22,36-37,41)^, home (n=3)^(22,25-26)^, in addition to a study carried out in a prison environment^(14)^ and one in a virtual environment^(27)^. The designs of analyzed studies, sample, country and target population sociodemographic characteristics are shown in Chart 1. The main variables assessed in the analyzed studies were knowledge, attitude, self-efficacy and Pap smear compliance.

The most common teaching strategies were participatory discussion sessions held in groups, in which women had the opportunity to ask questions and express personal issues related to CC prevention^(8-14,21,23,29,32,37,39,41,44-45)^. Other strategies used were lecture^(7-8,11-12,23,25,32,39,41,43)^, expository lesson^(9,11)^ and card games^(36)^.

Educational leaflets, or booklets, were the most used resources as support or complementary material, with the purpose of reinforcing the content^(34,39,42,44-45)^. These materials were usually delivered at the end of the educational activity^(14,21,24-26,31-32,35,37,39,42)^. Other visual resources were used, such as flipchart^(11,30,36-38)^, PowerPoint^(32-33)^ and posters^(31)^, in addition to audiovisual resources such as informative videos^(22-23,29-30,33-34,38,43)^, movies^(13,21,41,45)^, soap operas^(36)^, infographics^(38)^, role-play^(23)^, practical shows^(7,21,41)^ and telegram^(45)^.

Education through telephone interviews was also used in some studies to provide explanations about CC and its risks, the purpose of Pap smear test, the importance of correct frequency of testing, pre-examination care, in addition to information about returning to access post-exam result^(25,28,35)^.

Another strategy used was the PLNav, used to help women access health services through professionals from the same community who work, speak the same language and/or come from the same context as the target population. This format was applied in studies that demonstrated an increase in knowledge about CC screening and screening among participating women^(23,34,40)^.

The content of the educational activities used in the interventions addressed themes related to CC, including risk factors, ways of preventing the disease and possible symptoms, with emphasis on the importance of performing Pap smear. Moreover, many interventions addressed the benefits and barriers in relation to Pap smear, including knowledge, attitudes, beliefs and information about access to health services. Some activities also emphasized the relationship between CC and Human Papillomavirus (HPV) infection, healthy lifestyle behaviors including healthy nutrition, physical activity, and weight management.

Educational activities took place in a single session, in most of analyzed studies (n=14), lasting from 40 to 60 minutes^(8,11,23,25-26,28-29,33,35-38,40,43)^. In most studies, the educational activity was carried out in a group and delivered by a Community Health Worker (CHW)^(11,13,22-23,33,36-38,42)^. It should be noted that, in only four studies, the participation of nurses in delivering the educational intervention was explicit^(8,14,25,35)^.

Regarding the theoretical model used, the most frequent one as a basis for the intervention was the HBM, which aims to increase knowledge, improve attitudes, broaden the perception of the benefits of the test and health motivation, in addition to reducing the barriers to performing Pap smear, factors that can contribute to CC prevention^(7-8,10,13,21,26,31-32,35,42)^.

The Protective Motivation Theory was used in two studies and incorporates the cognitive process into a conceptual framework that describes intention and behavior^(24,39)^. Two studies used mapping principles, which consider the needs empirically observed in the target population^(33,36)^. In these studies, the intervention was applied by CHW^(33,36)^.

Studies have also used the Beliefs, Attitudes, Subjective Norms and Facilitating Factors (BASNEF) Model^(9)^, the SCT^(29,43)^, the Theory of Planned Behavior^(12)^, the PRECEDE-PROCEED Conceptual Model^(10)^ and the Fogg Behavior Model^(27)^, among others. Twelve articles included in this review used combined theoretical models^(11,14,22-23,30,34,36-38,40-41,44-45)^.

Study analysis showed that educational strategies, based on theories and culturally sensitive, can be used in combination for CC prevention, with emphasis on group discussions and lectures. Educational interventions that address knowledge about CC, barriers to screening and the importance of performing Pap smear seem to influence women’s health beliefs and behavior. Educational interventions that rely on the collaboration of CHW can contribute to the reduction of barriers to screening.

## DISCUSSION

CC is still common in women. This review analyzed thirty-three studies that used educational interventions aimed at CC prevention in women aged ≥18 years, in any context of care.

With technological advances, one can think of using different educational strategies to educate the population on a given topic. However, it is important to consider the available resources, the target population characteristics (e.g., educational level) and the feasibility of applying the technology. In the present review, the focus group sessions^(8-14,21,23,29,32,37,39,41,44-45)^ and educational leaflets were the most used strategies to promote prevention behaviors^(11,14,21,24-26,31-32,35,37,39,42)^, probably because their low cost makes them accessible and guarantees the delivery of the necessary educational content.

In the present research, the discussion sessions and the educational leaflets appeared combined with each other, or in a complementary way to other methodologies such as videos, lectures, telephone calls, among others. A systematic review that assessed the effects of educational interventions on CC screening behavior showed that the different methods used improved prevention behaviors among women^(46)^.

Studies that used video as an educational strategy showed a significant increase in CC knowledge. Participating women reported greater confidence in scheduling Pap smear, suggesting that video can be a potentially useful means of communicating health information^(22-23,29-30,33-34,38,43)^.

Phone call was also a strategy used to provide information about CC^(28,35)^ or as a form of invitation to women who did not initially respond to the screening test^(25)^. Consistent with a scoping review, telephone contact increased attendance at the health service for performing Pap smear^(47)^.

The content of the educational activities used in the analyzed studies is similar to the findings of a systematic review that concluded that the interventions mainly emphasize CC, Pap smear and HPV^(48)^. Effective educational programs targeting CC awareness need sophisticated and comprehensive planning and assessment of target audience needs, such as level of knowledge, beliefs, attitudes and behaviors.

In the present study, most participants were Latino or from rural communities. Latin women expressed better response to educational interventions; however, they were more likely to be diagnosed at an advanced stage of the disease, due to relatively low screening rates, poorer quality of life, and lack of health insurance^(49)^. Women from rural areas presented a number of barriers to obtaining health care, such as travel distance, transportation difficulties and access to specialized care^(49)^.

Studies using the HBM indicated that providing information on CC risk factors and early detection are crucial elements to increase the intention to perform CC screening and that educational interventions delivered by CHWs helped to reduce CC disparities among women. women from different backgrounds^(7-8,13,21,26,31-32,35,42)^.

Using educational booklets can be effective in increasing screening when the objective is to reach many women, with little or no knowledge about CC^(26)^. On the other hand, providing individual information over the phone, explaining the importance of the subject and making a personal invitation can be more effective strategies in reducing barriers^(26)^.

A study revealed a significant increase in screening rates in CHW-led interventions, which could be a useful approach as it uses trusted peer relationships to provide education and promote healthy behaviors, with an emphasis on CC prevention^(42)^.

The theoretical model most used by the studies included in this review was the HBM, similar to data observed in a systematic review and meta-analysis that showed that this model can be effective in promoting Pap smear compliance^(50)^.

### Study limitations

The present study presents as a limitation the lack of checking the references of analyzed studies to locate additional studies.

### Contributions to nursing

Educational interventions have the potential to increase knowledge, change health beliefs and reduce barriers to CC prevention. Theory-based and culturally sensitive interventions appear to have a positive impact on women’s health. This research revealed low participation of nurses in educational intervention programs and it is questioned whether the participation of this professional could contribute to reducing barriers to health prevention behaviors. This hypothesis should be tested in future studies.

## CONCLUSIONS

Study analysis showed that different educational strategies can be used in combination for CC prevention, with emphasis on group discussions and lectures. Educational leaflets were used as support material. The content of the interventions emphasized knowledge about CC, barriers to screening and the importance of performing Pap smear.

Most of studies indicated benefits of CHW’s performance and few had nurses as an intervenor. Studies that test theory-based, culturally sensitive educational interventions, with nurses as the intervenor, are needed to assess the impact of these interventions on women’s health.
